# Expression of netrin-1 receptors in retina of oxygen-induced retinopathy in mice

**DOI:** 10.1186/1471-2415-14-102

**Published:** 2014-08-22

**Authors:** Dan Liu, Si-Qi Xiong, Lei Shang, Xiao-feng Tian, Jing Yang, Xiao-Bo Xia

**Affiliations:** 1 Department of Ophthalmology, Xiangya Hospital, Central South University, Changsha, Hunan 410008, China; 2 Department of Anatomy and Neurobiology, School of Basic Medical Sciences, Central South University, Changsha, Hunan 410013, China

**Keywords:** Netrin-1, UNC5B, Neogenin, Retinal neovascularization

## Abstract

**Background:**

Netrin-1 has been reported to promote retinal neovascularization in oxygen-induced retinopathy (OIR). However, netrin-1 receptors, which may mediate netrin-1 action during retinal neovascularization, have not been characterized. In this study, we investigated netrin-1 receptor subtype expression and associated changes in the retinas of mice with OIR.

**Methods:**

C57BL/6J mice were exposed to 75±2% oxygen for 5 days and then returned to normal air to induce retinal neovascularization. Reverse transcriptase polymerase chain reaction (RT-PCR) and Western blot were used to examine the expression of netrin-1 receptor subtypes in the mouse retinas. Double staining of netrin-1 receptor subtypes and isolectin B4 was used to determine the location of the netrin-1 receptor subtypes in the retinas. Inhibition of retinal neovascularization was achieved by UNC5B shRNA plasmid intravitreal injection. Retinal neovascularization was examined by fluorescein angiography and quantification of preretinal neovascular nuclei in retinal sections.

**Results:**

RT-PCR results showed that, except for UNC5A, netrin-1 receptor subtypes UNC5B, UNC5C, UNC5D, DCC, neogenin, and A2b were all expressed in the retinas of OIR mice 17 days after birth. Western blots showed that only UNC5B expression was significantly increased on that day, and immunofluorescence results showed that only UNC5B and neogenin were expressed in retinal vessels. Treatment of OIR mice with the UNC5B shRNA plasmid dramatically reduced neovascular tufts and neovascular outgrowth into the inner limiting membrane.

**Conclusions:**

UNC5B may promote retinal neovascularization in OIR mice.

## Background

Netrin-1 has been reported to stimulate proliferation and migration of endothelial cells *in vitro*, and promote developmental and pathological angiogenesis *in vivo*[1,2]. To investigate the role of netrin-1 in retinal angiogenesis, we characterized the expression of netrin-1 in the retinas of mice with oxygen-induced retinopathy (OIR) [3], and injected netrin-1 shRNA into the vitreous cavity of OIR mice [4]. We showed that netrin-1 expression was simultaneously elevated with an increase of retinal neovascularization. Intravitreal injection of netrin-1 shRNA effectively inhibited retinal neovascularization, indicating that netrin-1 may promote retinal angiogenesis in OIR mice.

In mammals, receptors of netrin-1 include the DCC family (deleted in colorectal cancers) and the UNC5 homologs. Together, the receptors include DCC, neogenin, UNC5A, UNC5B, UNC5C, and UNC5D. A2b was also reported to be a netrin-1 receptor [5]. As an axonal guidance cue, netrin-1 attracts or repels axonal growth, depending on which receptor subtype it recognizes. DCC mediated chemoattraction [6], while repulsion required a UNC5 homolog, acting alone or together with DCC family receptors [7,8]. However, whether netrin-1 receptors are expressed in the retinas of OIR mice, and which subtypes are involved in retinal neovascularization during this expression, are presently unknown. The current study was designed to characterize these receptors and subtypes. We have developed an OIR model in mice, and have characterized the expression of the netrin-1 receptors UNC5A-D, DCC, neogenin, and A2b in the retinas of normal and OIR mice. We observed that increased levels of UNC5B correlated with retinal angiogenesis in OIR mice, and the expressions of UNC5B and neogenin were located in new OIR vessels. Based upon these observations, UNC5B and/or neogenin may be involved in retinal neovascularization. We then injected a UNC5B shRNA plasmid into the vitreous cavity of OIR mice, and observed that the inhibition of retinal neovascularization, consistent with UNC5B, may promote retinal neovascularization in these mice.

## Methods

### Animal model

C57BL/6 J mice were available from the animal center of Central South University, were used in the present study. IR was produced as described by Smith et al. [9]. Seven-day-old mice were exposed to 75 ± 5% oxygen for 5 days, together with their nursing mothers. At postnatal day 12 (P12), the animals were returned to room air (21% oxygen). The mice were then exposed to 12 hours of cyclical broad spectrum light. The room temperature was maintained at 23°C - 28°C. For biochemical studies, mice were sacrificed by neck dislocation, and for histological analyses, mice were sacrificed by hyper-anesthesia. All experimental procedures used in the present study were approved by Ethics Committee of Xiangya School of Medicine, in accordance with the NIH guidelines for use and care of laboratory animals.

### Assessment of retinal vascularization

Angiography with high-molecular-weight fluorescein-dextran was performed on P17. Normal and OIR mice were anesthetized using intraperitoneal injection of pentobarbital sodium, and perfused through the left ventricle with phosphate-buffered saline (PBS) containing 1 mL 50 g/L fluorescein-labeled high-molecular-weight (2 million) dextran (Sigma-Aldrich, St. Louis, MO, USA). Subsequently, the eyes were enucleated and fixed in 40 g/L paraformaldehyde for 10 minutes. The retinas were dissected and placed in 40 g/L paraformaldehyde for 10 minutes, then flat-mounted using a 1:1 solution of glycerol and PBS.

### Histological analysis of neovascularization

On P17, the eyes of normal and OIR mice were enucleated, fixed with 40 g/L paraformaldehyde in PBS overnight at 4°C, then embedded in paraffin. Serial cross sections (5 μm) of the whole eye were stained with hematoxylin and eosin to visualize the cell nuclei. Ten non-serial sections, except sections containing the optic nerve, were analyzed per eye. The nuclei of new vessels extending from the retina to the vitreous were counted in 60 sections in each group.

### Immunofluorescent staining of netrin-1 receptor subtypes

Eyeballs were removed following transcardiac perfusion with 4% paraformaldehyde in PBS at certain survival time points, then postfixed in perfusion solution overnight, followed by cryoprotection in 30% sucrose. Sagittal frozen sections of retina were cut to a thickness of 12 μm, and mounted on glass slides. The retinal sections were covered with 5% BSA for 2 hours at room temperature to reduce nonspecific background staining. To identify the expression of receptor subtypes in vascular endothelial cells, double staining to detect UNC5B-D, DCC, and neogenin with isolectin B4 (an endothelial cell marker) was performed. The retinal sections were incubated with the primary antibodies, which were diluted in PBS containing 3% bovine serum albumin (BSA), overnight at 4°C. The primary antibodies included goat polyclonal anti-UNC5B (AF1006 1:50, R&D Systems, MN, USA), goat polyclonal anti-UNC5C (SC-54441 1:100, Santa Cruz Biotechnology, CA, USA), rabbit polyclonal anti-UNC5D (SC-135262 1:100, Santa Cruz Biotechnology), rabbit polyclonal anti-neogenin (SC-15337 1:100, Santa Cruz Biotechnology), rabbit polyclonal anti-DCC (SC-35184 1:100, Santa Cruz Biotechnology), rabbit polyclonal anti-A2b (SC-28996 1:100, Santa Cruz Biotechnology), and mouse anti-GAPDH (Mab-2005079 1:8000, ProMab, CA, USA).

The sections were rinsed with PBS three times and incubated with 1:300 Cy3-conjugated secondary (Sigma-Aldrich) and 1:400 Alexa 488-conjugated antibody (Invitrogen, Grand Island, NY, USA) for 2 hours at room temperature. Control sections were prepared by incubating with the BSA mixture without the primary antibody. Sections were counterstained with Hoechst 33342 (1:50, Sigma-Aldrich), washed, and mounted with anti-fading medium, before microscopy examination. Digital images were obtained using fluorescence microscopy (BH-40; Olympus, Tokyo, Japan). The immunofluorescence staining procedure was repeated three times.

### RT-PCR analysis of netrin-1 receptor mRNA expression

Expressions of UNC5A-D, DCC, and neogenin mRNA were determined using reverse transcriptase polymerase chain reaction (RT-PCR). We used brain tissue as a positive control. Retinas were lysed in TRIzol, and RNA was extracted and purified without any contaminating genomic DNA, according to the manufacturer’s instructions (Invitrogen). The first strand of cDNA was transcribed from 2 μg total RNA using oligo(dT) (Invitrogen). The first strand with cDNA not added was used as a negative control and generated using Moloney murine leukemia virus transcriptase (Invitrogen). PCR was performed using the following primer pairs:

A2b, 5′-CTACTTTCTGGTATCCCTGG-3′ and 5′-CTCGGTTCCTGTAGGCATAG-3′;

DCC, 5′-AACGCTGTCTGTGGACCGAG-3′ and 5′-GTTGCTTCATTAGCCCTTCC-3′;

neogenin, 5′-TCAGATGATCGACGCCAGCT-3′ and 5′-GTCCCAGCATCATCCTCAGT-3′;

UNC5A, 5′-GCTTCCAGCCTGTCAGCATC-3′ and 5′-AGAGCATCGTGGGTGTCGTG-3′;

UNC5B, 5′-GGGCACGTACCCAGGCGATT-3′ and 5′-CGAAGTAGTTTAGGTACCGGTCC-3′;

UNC5C, 5′-TAACCTGAAGAACCAGAGCC-3′ and 5′-AGGGTCCAGGAGAGGTAAGT-3′;

UNC5D, 5′-ATTGAGAATGCCAGCGACAT-3′ and 5′-TGTCCACACAGTAAACTCTC-3′; and

tubulin, 5′-GCTTCAAGGTTGGCATCAAC-3′ and 5′-TAGTATTCCTCTCCTTCTTC-3′.

Amplification was performed with a cycle (94°C/5 minutes), (94°C/45 seconds, T_m_/45 seconds, 72°C/45 seconds) × 32 cycles, 72°C/10 minutes. The T_m_ of each receptor was UNC5A, 56°C; UNC5B, 56°C; UNC5C, 5°C; UNC5D, 52°C; A2b, 60°C; DCC, 60°C; and neogenin, 52°C.

### Western blot analysis of the netrin-1 receptor protein

For biochemical studies, the eyeballs were removed after neck dislocation on P12, P17, and P21, and the animals with weight >6 g were used. The retinas were dissected and placed in cold PBS on ice, carefully soaked with a piece of filter paper, and collected in 1.5-ml test tubes and weighed. They were dissected and lysed in radioimmunoprecipitation assay (RIPA) lysis buffer (Pierce, Rockford, IL, USA). Protein concentrations were determined with a protein assay reagent (Pierce, Rockford, IL, USA), using the bicinchoninic acid assay method. For each sample, 100 μg protein was fractionated using 10% sodium dodecyl sulfate polyacrylamide gel electrophoresis, and transferred onto polyvinylidene membranes (Pierce). The membranes were incubated with anti-UNC5B (1:100), anti-UNC5C (1:100), anti-UNC5D (1:100), anti-DCC (1:50), anti-A2b(1:100), or anti-neogenin (1:100) for 2 hours at room temperature, then incubated with a specific secondary antibody (horseradish peroxidase-conjugated anti-immunoglobulin G). Binding was detected using an enhanced chemiluminescence (ECL) method.

Working solutions for the ECL method were prepared according to the manufacturer’s instructions, and added to the membranes for 1 minute. The membranes were removed from the solutions and placed in plastic sheet protectors. Each membrane was exposed to CL-Xposure film (Pierce) for 90 seconds. The experiments were repeated three times. Band intensities were quantified with BandScan software Version 4.5 (Glyko Software, Novato, CA, USA). Levels of glyceraldehyde 3-phosphate dehydrogenase (GAPDH) production were used for standardization. Results are expressed as the ratio of specific protein to GAPDH production.

### Intravitreal injection for UNC5B shRNA infection

On P12, ten mice were anesthetized by intraperitoneal injection of 10% chloral hydrate (30 mg/kg body weight). Intravitreal injections were performed using a 32-gauge Hamilton needle and syringe. Among the 10 mice, one eye was intravitreally injected with 1 μl of the UNC5B shRNA plasmid (Santa Cruz Biotechnology, sc-61847-SH), and the other eye, as the negative control, received an intravitreal injection with 1 μl of the control shRNA plasmid-A (Santa Cruz Biotechnology, sc-108060). To assess the angiostatic effect of UNC5B shRNA on retinal neovascularization, fluorescein angiography and quantification of preretinal neovascular nuclei in retinal sections were performed on P17.

### Statistical analysis

All data were expressed as the mean ± SD. Student’s *t*-test was used to compare groups. Significance was set at p < 0.05.

## Results

### Analysis of retinal neovascularization

**Figure 1:**
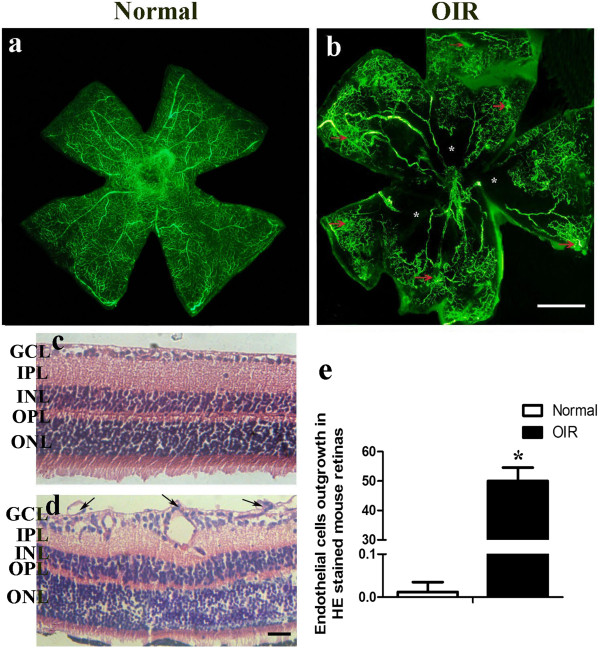
**Assessment of retinal vascularization. (a, b)** Angiographic analysis by fluorescein-dextran. **(c, d)** Histological demonstration of retinal neovascularization. **(a, c)** Normal mice. **(e)** Quantification of retinal neovascularization. **(b, d)** Oxygen-induced retinopathy (OIR) mice. **(a)** Normal mice, retinal vessels form a polygonal reticular pattern. **(b)** OIR mice, neovascular tufts appear as hyperfluorescence at the junction between the perfused and nonperfused areas (indicated by red arrows). The nonperfused areas are marked with an asterisk. **(c)** Normal mice, no neovascular cell nuclei extend beyond the internal limiting membrane. **(d)** OIR mice, many neovascular cell nuclei protrude into the vitreous cavity (indicated by arrows). GCL, ganglion cell layer; IPL, inner plexiform layer; INL, inner nuclear layer; OPL, outer plexiform layer; ONL, outer nuclear layer. Bar = 50 μm.

Fluorescence angiography was used to visualize the retinal vasculature of normal and OIR mice. Retinas from normal mice had both superficial and deep vascular layers, which extended from the optic nerve to the periphery. Vessels in the superficial retinal layer formed a fine radial branching pattern. In deep retinal layers, the vessels formed a polygonal reticular pattern (Figure 1a). Retinas from OIR mice were characterized by central nonperfused areas and neovascular tufts (Figure 1b). Serial paraffin cross sections were used to count the number of neovascular nuclei on the vitreal side of the internal limiting membrane. In OIR mouse retinas, many vascular cell nuclei extended beyond the internal limiting membrane (Figure 1d,e), and the average number of neovascular nuclei per section was 50.70 ± 4.56, while there were very few neovascular nuclei in the normoxic control mice (Figure 1c,e).

### RT-PCR results

**Figure 2:**
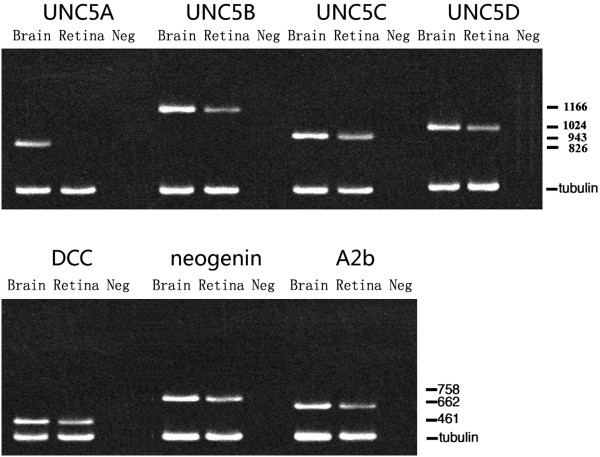
**RT-PCR analysis of netrin-1 receptor mRNA in retinas of OIR mice.** The expressions of UNC5B, UNC5C, UNC5D, neogenin, A2b, and DCC are all detected in retinas of OIR mice, while UNC5A is not detected.

Brain tissue was used as a positive control, and no RNA was used as a negative control. Except UNC5A, we determined that netrin-1 receptors, UNC5B, UNC5C, UNC5D, neogenin, A2b, and DCC were all expressed in the retinas of OIR mice on P17 (Figure 2).

### Western blots

**Figure 3:**
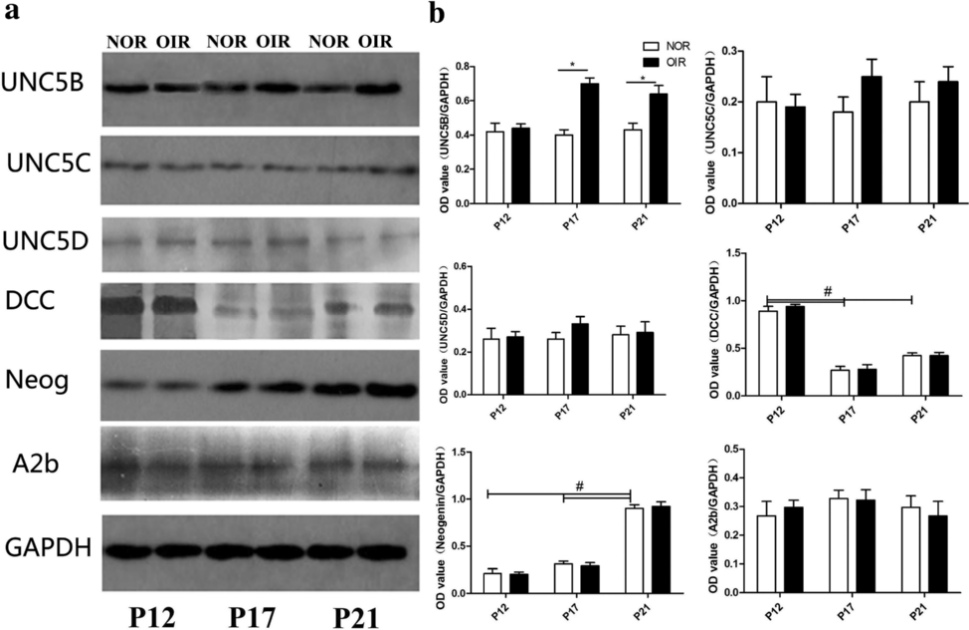
**Expression of UNC5B-D, DCC, neogenin, and A2b in retinas of normal and OIR mice on P12, P17, and P21. (a)** Expression of UNC5B-D, DCC, neogenin, and A2b in normal and OIR mice. **(b)** Relative grayscale of UNC5B-D, DCC, neogenin, A2b, and GAPDH in normal and OIR groups. The UNC5B level of the OIR group is increased significantly compared with the normal group on P17 and P21 (p < 0.05), while the expressions of UNC5C, UNC5D, neogenin, A2b, and DCC show no significant differences between the normal and OIR mice (p > 0.05). In normal mice, DCC expression is greater on P12 than P17 and P21 (p < 0.05), and neogenin expression increases (p < 0.05) from P12 to P21.

Compared with normal mice, expression of all the receptors showed no differences in the OIR mice on P12 (Figure 3, p > 0.05). The expression of UNC5B in the retina of OIR mice was significantly upregulated, compared with normal mice, on P17 and P21 (Figure 3, p < 0.05), while the expression of UNC5C, UNC5D, neogenin, A2b, and DCC showed no difference between normal and OIR mice (Figure 3, all p > 0.05). In normal mice, DCC expression was greater on P12 than on P17 and P21 (Figure 3, p < 0.05). There was no difference in expression between P17 and P21 (Figure 3, p > 0.05), while neogenin expression increased (Figure 3, p < 0.05) from P12 to P21. Last, the expressions of UNC5B, UNC5C, UNC5D, and A2b showed no differences on P12, P17, and P21 in normal mice (Figure 3, p > 0.05).

### Staining of netrin-1 receptors in retina

**Figure 4:**
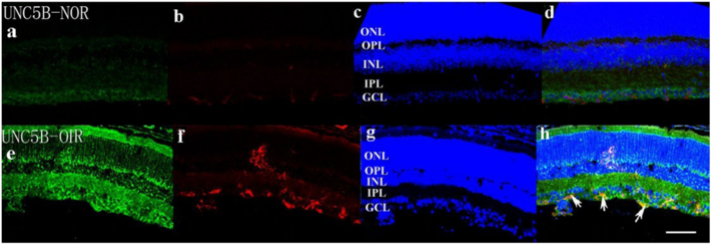
**Double immunostaining of UNC5B with isolectin B4.** UNC5B is expressed in the ganglion cell layer, inner plexiform layer, and outer plexiform layer, and the expression of UNC5B is elevated in OIR mice compared with normal mice. (**a**, **e**; green). Endothelial cells increase more in OIR mice than in normal mice (**b**, **f**; red). Hoechst staining in normal and OIR mice (**c**, **g**, blue). UNC5B colocalizes with isolectin B4 in OIR mice (**d**, **h**; yellow, indicated by arrows). GCL, ganglion cell layer; IPL, inner plexiform layer; INL, inner nuclear layer; OPL, outer plexiform layer; ONL, outer nuclear layer. Bar = 50 μm.

**Figure 5:**
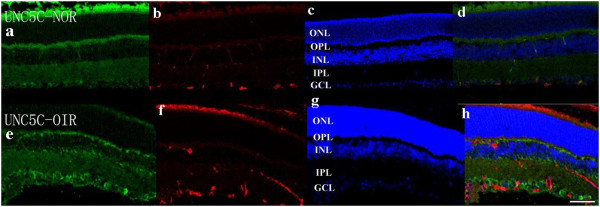
**Double immunostaining of UNC5C with isolectin B4.** UNC5C is expressed in the ganglion cell layer, inner plexiform layer, and outer plexiform layer in normal and OIR mice (**a**, **e**, green). There are more endothelial cells in OIR mice than in normal mice (**b**, **f**, red). Hoechst staining in normal and OIR mice (**c**, **g**, blue). UNC5C did not colocalize with isolectin B4 in OIR mice **(d,h)**. GCL, ganglion cell layer; IPL, inner plexiform layer; INL, inner nuclear layer; OPL, outer plexiform layer; ONL, outer nuclear layer. Bar = 50 μm.

**Figure 6:**
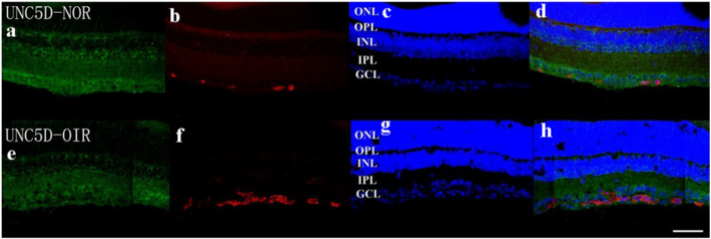
**Double immunostaining of UNC5D with isolectin B4.** UNC5D is expressed in the ganglion cell layer, inner plexiform layer, and outer plexiform layer in normal and OIR mice. (**a**, **e**, green). There are more endothelial cells in OIR mice than in normal mice (**b**, **f**, red). Hoechst staining in normal and OIR mice (**c**, **g**, blue). UNC5D does not colocalize with isolectin B4 in OIR mice **(d, h)**. GCL, ganglion cell layer; IPL, inner plexiform layer; INL, inner nuclear layer; OPL, outer plexiform layer; ONL, outer nuclear layer. Bar = 50 μm.

**Figure 7:**
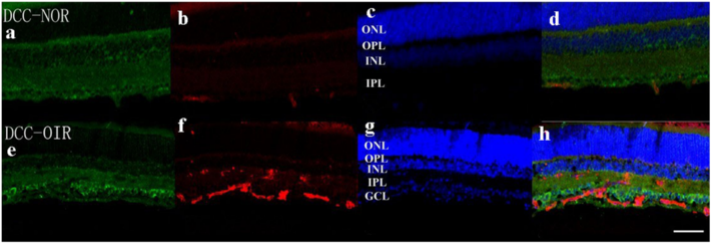
**Double immunostaining of DCC with isolectin B4.** DCC is expressed in the ganglion cell layer, inner plexiform layer, and outer plexiform layer in normal and OIR mice. (**a**, **e**, green). There are more endothelial cells in OIR mice than in normal mice (**b**, **f**, red). Hoechst staining in normal and OIR mice (**c**, **g**, blue). DCC does not colocalize with isolectin B4 in OIR mice **(d, h)**. GCL, ganglion cell layer; IPL, inner plexiform layer; INL, inner nuclear layer; OPL, outer plexiform layer; ONL, outer nuclear layer. Bar = 50 μm.

**Figure 8:**
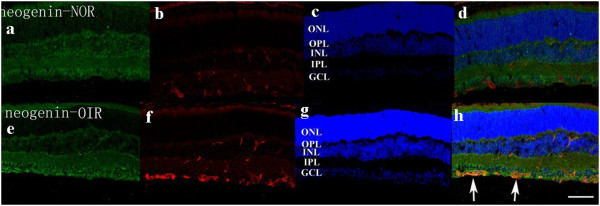
**Double immunostaining of neogenin with isolectin B4.** Neogenin is expressed in the ganglion cell layer, inner plexiform layer, and outer plexiform layer in normal and OIR mice (**a**, **e**, green). There are more endothelial cells in OIR mice than in normal mice (**b**, **f**, red). Hoechst staining in normal and OIR mice (**c**, **g**, blue). Neogenin colocalizes with isolectin B4 in OIR mice (**d**, **h**; yellow, indicated by arrows). GCL, ganglion cell layer; IPL, inner plexiform layer; INL, inner nuclear layer; OPL, outer plexiform layer; ONL = outer nuclear layer. Bar = 50 μm.

**Figure 9:**
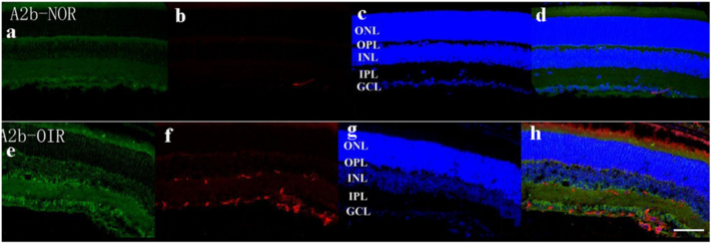
**Double immunostaining of A2b with isolectin B4.** A2b is expressed in the ganglion cell layer, inner plexiform layer, and outer plexiform layer in normal and OIR mice (**a**, **e**, green). There are more endothelial cells in OIR mice than in normal mice (**b**, **f**, red). Hoechst staining in normal and OIR mice (**c**, **g**, blue). A2b does not colocalize with isolectin B4 in OIR mice **(d, h)**. GCL, ganglion cell layer; IPL, inner plexiform layer; INL, inner nuclear layer; OPL, outer plexiform layer; ONL, outer nuclear layer. Bar = 50 μm.

Confocal microscopy of double immunostaining showed that UNC5B, UNC5C, UNC5D, neogenin, A2b, and DCC were expressed in the ganglion cell layer, inner plexiform layer, and outer plexiform layer of retinas. There was no significant difference among the six receptor subtypes (Figures 4, 5, 6, 7, 8, 9, green). However, isolectin B4 staining (an endothelial cell marker) was greater in OIR than in normal mice (Figures 4, 5, 6, 7, 8, 9, red), and only UNC5B and neogenin were colocalized with isolectin B4 in OIR mice (Figures 4 and 8, yellow, indicated by arrows).

### Suppression of UNC5B expression by UNC5B shRNA in OIR mice

**Figure 10:**
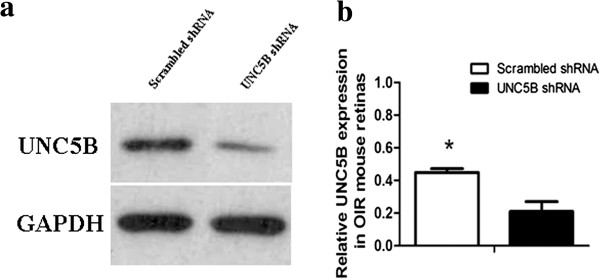
**Western blot analysis of the effects of UNC5B shRNA on UNC5B expression in murine retinas.** Representative western blot **(a)** and relative expression of UNC5B in murine retinas **(b)**. UNC5B expression is significantly decreased in the retinas of OIR mice transfected with UNC5B shRNA, compared with scrambled shRNA on P17 (p < 0.01).

The effects of targeting UNC5B with RNAi on UNC5B expression were investigated by infection of UNC5B shRNA or scrambled shRNA control in retinas of OIR mice. Compared with scrambled shRNA injection, intravitreal injection of UNC5B shRNA on P12 resulted in downregulation of UNC5B expression in the retinas of OIR mice on P17 (Figure 10).

### Suppression of retinal neovascularization by the UNC5B shRNA plasmid

**Figure 11:**
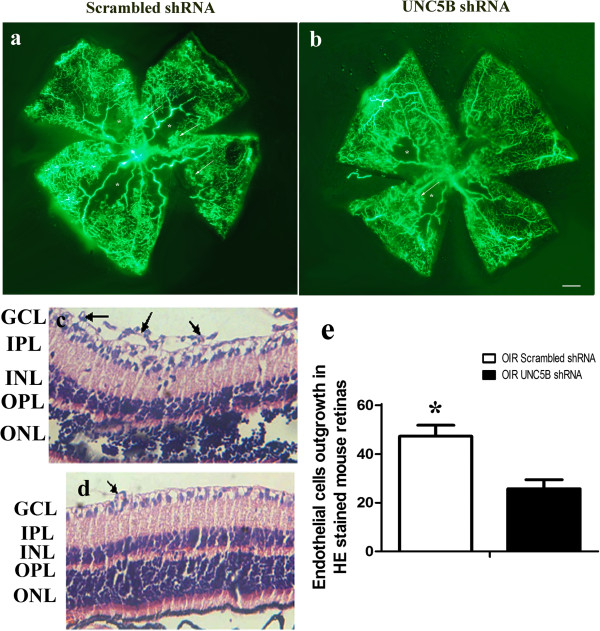
**Inhibition of ischemia-induced retinal neovascularization by UNC5B shRNA. (a, c)** Retinas transfected with scrambled shRNA. **(b, d)** Retinas transfected with UNC5B shRNA. **(e)** Quantification of retinal neovascularization. **(a, b)** Angiographic analysis by fluorescein-dextran, the numbers of neovascular tufts (marked by white arrows) are reduced in the retinas transfected with UNC5B shRNA. **(c,d)** Histological demonstration of retinal neovascularization, UNC5B shRNA infection results in approximately a 50% reduction of preretinal neovascularization. GCL, ganglion cell layer; IPL, inner plexiform layer; INL, inner nuclear layer; OPL, outer plexiform layer; ONL, outer nuclear layer. Bar = 50 μm.

To determine the angiostatic effect of UNC5B shRNA plasmid on ischemia-induced retinal neovascularization, fluorescein angiography was performed on OIR mice on P17. Retinas of OIR mice transfected with scrambled shRNA had multiple neovascular tufts (hyperfluorescence, Figure 11a). In contrast, fewer neovascular complexes were observed in the retinas of OIR mice transfected with the UNC5B shRNA plasmid (Figure 11b). To further confirm the inhibitory effects of UNC5B shRNA on angiogenesis, retinal neovascularization was assessed histologically by counting the neovascular nuclei protruding into the vitreous cavity (Figure 11c,d). The average number of neovascular nuclei extending beyond the internal limiting membrane per section was 47.32 ± 4.05 in OIR mice transfected with UNC5B shRNA and 25.76 ± 3.68 in OIR mice injected with scrambled shRNA, thus approximately representing a 50% reduction in retinal neovascularization.

## Discussion

In OIR mice, retinal neovascularization formed after P14 and reached a maximum at P17 and P21 [9], so we detected netrin-1 receptor expression on P12 (neovascularization not formed yet), P17, and P21 (neovascularization peaked). Compared with normal mice, the expression of UNC5B did not show statistical significance in the OIR mice on P12, but increased significantly on P17 and P21. Our results showed a positive correlation between UNC5B overexpression and retinal neovascularization. For the other receptors, there were no significant differences between the normal and OIR mice on P12, P17 and P21.

Our immunohistochemistry results showed that UNC5B, UNC5C, UNC5D, DCC, A2b, and neogenin were all expressed in the ganglion cell layer, inner plexiform layer, and outer plexiform layer, but only UNC5B and neogenin were expressed in the retinal vessels of OIR mice. Regarding the seven netrin-1 receptors, our study showed that UNC5B and/or neogenin may have been implicated in retinal neovascularization.

Among the netrin-1 receptors, UNC5B appeared to be the best receptor candidate to mediate the angiogenic effects of netrin-1. UNC5B was expressed in developing blood vessels in murine embryos, and controlled morphogenesis of the vascular system [10]. In addition to being expressed in embryos, recent studies showed that UNC5B was also expressed during postnatal and pathological angiogenesis [11]. In the study by Larrive et al. [11], UNC5B was expressed in sprouting angiogenesis induced by OIR, matrigel, or tumor implantation, suggesting that UNC5B may be required during sprouting angiogenesis.

In our study, double staining of UNC5B and isolectin B4 in cross sections showed that UNC5B was expressed in the endothelial cells of newly formed retinal vessels in OIR mice. This result confirmed the conclusions of Larrive et al. [11], and indicated that UNC5B may be required for retinal neovascularization. Furthermore, in the current study, we showed that expression of UNC5B was significantly elevated on P17, compared with normal age-matched mice. At the same time, new vessels were formed in OIR mice, so there was a significant positive correlation between UNC5B overexpression and retinal neovascular formation. Because the expression of UNC5B was localized to the sprouting vessels and was significantly elevated during the active angiogenic period of the OIR mice, we postulated a role of UNC5B in hypoxia-driven neovascularization. In our previous studies [3,11], we detected the overexpression of netrin-1 in OIR mice on P17, so there was also a positive correlation between netrin-1 overexpression and UNC5B overexpression in OIR mice on P17, suggesting that UNC5B mediated netrin-1 action during retinal neovascularization.

However, the possible role of UNC5B in angiogenesis has been controversial. Some studies [10,11] suggested that UNC5B was a repulsive netrin-1 receptor in endothelial cells, controlling morphogenesis of the vascular system. Activation of UNC5B could therefore inhibit developmental and pathological angiogenesis. However, others [12,13] reported that UNC5B was a proangiogenic factor for vessels *in vivo* and *in vitro*. Navankasattusas et al. [12] observed that umbilical arteries isolated from UNC5B-deficient embryos were unable to support vessel outgrowth *in vitro*, and deletion of UNC5B in endothelial cells in mice led to reduction of placental arterioles. Epting et al. [13] reported that netrin-1 and its receptor, UNC5B, were upstream regulators of ELMO1/DOCK180 in endothelial cells, leading to Rac1 activation *in vitro*, which has been reported to be necessary for vascular development [14]. To reconcile this controversy, Castets et al. [15] reported that unbound UNC5B acted in a proapoptotic manner in endothelial cells, while netrin-1 bound to UNC5B acted as a survival signal for endothelial cells, provoking a proangiogenic response. However, whether UNC5B promoted or inhibited retinal neovascularization of OIR mice was still unknown. To further investigate the role of UNC5B in retinal neovascularization, we observed the effects of inhibition of retinal angiogenesis by RNA interference (RNAi) of UNC5B in the OIR mouse model. As shown in the present study, UNC5B expression decreased dramatically following UNC5B shRNA infection, and retinal neovascularization was clearly reduced by UNC5B shRNA infection. Together, these results showed that UNC5B functioned as a proangiogenic growth factor in the pathological neovascularization of OIR mice. Therefore, UNC5B could be a novel target for the therapy of diabetic retinopathy, retinopathy of prematurity, and other ocular neovascular diseases.

Neogenin is another receptor subtype of netrin-1 implicated in angiogenesis. A previous study [1] reported that neogenin was expressed in vascular smooth muscle cells (VSMCs), showing that it was responsible for cell migration and proliferation of VSMCs mediated by netrin-1. Lejmi et al. [16] reported that neogenin was upregulated in vascular endothelial growth factor-stimulated endothelial cells, choroidal neovessels, and tumor angiogenesis, while silencing of either neogenin or UNC5B abolished netrin-4′s inhibitory effect on endothelial cell migration, choroidal neovascularization, and tumor angiogenesis.

Consistent with these studies, we also observed that neogenin was expressed in the newly formed retinal vessels. This result may indicate a role of neogenin in retinal neovascularization, although neogenin expression was not elevated during the active angiogenic period of OIR mice. However, in addition to being a receptor for netrin-1, neogenin is also a receptor for the repulsive guidance molecule (RGM) families. While netrin-1–neogenin interactions resulted in a chemoattractive axon guidance response, the interaction between neogenin and RGM induced a chemorepulsive response [17]. Thus, further studies are needed to determine whether neogenin binds to netrin-1 or RGM in retinas of OIR mice, and whether neogenin participates in retinal neovascularization.

In the present study, we did not detect UNC5A in the retinas of OIR mice on P17. In addition, although we observed the expression of UNC5C, UNC5D, DCC, and A2b in the retinas of OIR mice, the expressions of these receptors were not significantly different between the OIR mice and normal mice. They were also not detected in newly formed vessels by immunofluorescent staining, suggesting that these receptors may not be involved in retinal neovascularization in OIR mice.

## Conclusions

UNC5B may promote retinal neovascularization in OIR mice, and may be a novel target for the therapy of ocular neovascular diseases.

## Competing interests

The authors declare that they have no competing interests.

## Authors’ contributions

DL and X-BX designed the experiments; DL and S-QX performed the experiments; LS, X-FT, and JY analyzed the data; DL and S-QX drafted the manuscript; LS and X-FT revised the manuscript and participated in revisions; X-BX revised the manuscript for English writing; and all authors participated in critical revision of the manuscript and approval of the final manuscript.

## Pre-publication history

The pre-publication history for this paper can be accessed here:

http://www.biomedcentral.com/1471-2415/14/102/prepub

## References

[B1] Park KW Crouse D Lee M Karnik SK Sorensen LK Murphy KJ Kuo CJ Li DY The axonal attractant Netrin-1 is an angiogenic factor Proc Natl Acad Sci U S A 2004 101 46 16210 16215 10.1073/pnas.0405984101 15520390 PMC528958

[B2] Wilson BD Ii M Park KW Suli A Sorensen LK Larrieu-Lahargue F Urness LD Suh W Asai J Kock GA Thomas KR Chien C-B Losordo DW Li DY Netrins promote developmental and therapeutic angiogenesis Science 2006 313 5787 640 644 10.1126/science.1124704 16809490 PMC2577078

[B3] Tian XF Xia XB Xiong SQ Jiang J Liu D Liu JL Netrin-1 overexpression in oxygen-induced retinopathy correlates with breakdown of the blood-retina barrier and retinal neovascularization Ophthalmologica 2011 226 2 37 44 10.1159/000324474 21508652

[B4] Xu H Liu J Xiong S Le YZ Xia X Suppression of retinal neovascularization by lentivirus-mediated netrin-1 small hairpin RNA Ophthalmic Res 2012 47 3 163 169 10.1159/000331428 22122983 PMC3251241

[B5] Moore SW Tessier-Lavigne M Kennedy TE Netrins and their receptors Adv Exp Med Biol 2007 621 17 31 10.1007/978-0-387-76715-4_2 18269208

[B6] Fazeli A Dickinson SL Hermiston ML Tighe RV Steen RG Small CG Stoeckli ET Keino-Masu K Masu M Rayburn H Simons J Bronson RT Gordon JI Tessier-Lavigne M Weinberg RA Phenotype of mice lacking functional Deleted in colorectal cancer (Dcc) gene Nature 1997 386 6627 796 804 10.1038/386796a0 9126737

[B7] Hong K Hinck L Nishiyama M Poo MM Tessier-Lavigne M Stein E A ligand-gated association between cytoplasmic domains of UNC5 and DCC family receptors converts netrin-induced growth cone attraction to repulsion Cell 1999 97 7 927 941 10.1016/S0092-8674(00)80804-1 10399920

[B8] Keleman K Dickson BJ Short- and long-range repulsion by the Drosophila Unc5 netrin receptor Neuron 2001 32 4 605 617 10.1016/S0896-6273(01)00505-0 11719202

[B9] Smith LE Wesolowski E McLellan A Kostyk SK D’Amato R Sullivan R D’Amore PA Oxygen-induced retinopathy in the mouse Invest Ophthalmol Vis Sci 1994 35 1 101 111 7507904

[B10] Lu X Le Noble F Yuan L Jiang Q De Lafarge B Sugiyama D Breant C Claes F De Smet F Thomas JL Autiero M Carmeliet P Tessier-Lavigne M Eichmann A The netrin receptor UNC5B mediates guidance events controlling morphogenesis of the vascular system Nature 2004 432 7014 179 186 10.1038/nature03080 15510105

[B11] Larrivee B Freitas C Trombe M Lv X Delafarge B Yuan L Bouvree K Breant C Del Toro R Brechot N Germain S Bono F Dol F Claes F Fischer C Autiero M Thomas JL Carmeliet P Tessier-Lavigne M Eichmann A Activation of the UNC5B receptor by Netrin-1 inhibits sprouting angiogenesis Genes Dev 2007 21 19 2433 2447 10.1101/gad.437807 17908930 PMC1993874

[B12] Navankasattusas S Whitehead KJ Suli A Sorensen LK Lim AH Zhao J Park KW Wythe JD Thomas KR Chien CB Li DY The netrin receptor UNC5B promotes angiogenesis in specific vascular beds Development 2008 135 4 659 667 10.1242/dev.013623 18223200 PMC2612632

[B13] Epting D Wendik B Bennewitz K Dietz CT Driever W Kroll J The Rac1 regulator ELMO1 controls vascular morphogenesis in zebrafish Circ Res 2010 107 1 45 55 10.1161/CIRCRESAHA.109.213983 20466982

[B14] Tan W Palmby TR Gavard J Amornphimoltham P Zheng Y Gutkind JS An essential role for Rac1 in endothelial cell function and vascular development FASEB J 2008 22 6 1829 1838 10.1096/fj.07-096438 18245172

[B15] Castets M Coissieux MM Delloye-Bourgeois C Bernard L Delcros JG Bernet A Laudet V Mehlen P Inhibition of endothelial cell apoptosis by netrin-1 during angiogenesis Dev Cell 2009 16 4 614 620 10.1016/j.devcel.2009.02.006 19386270

[B16] Lejmi E Leconte L Pedron-Mazoyer S Ropert S Raoul W Lavalette S Bouras I Feron JG Maitre-Boube M Assayag F Feumi C Alemany M Jie TX Merkulova T Poupon MF Ruchoux MM Tobelem G Sennlaub F Plouët J Netrin-4 inhibits angiogenesis via binding to neogenin and recruitment of Unc5B Proc Natl Acad Sci U S A 2008 105 34 12491 12496 10.1073/pnas.0804008105 18719102 PMC2518829

[B17] Cole SJ Bradford D Cooper HM Neogenin: a multi-functional receptor regulating diverse developmental processes Int J Biochem Cell Biol 2007 39 9 1569 1575 10.1016/j.biocel.2006.11.009 17204444

